# Supplementary material for: Conditional chemoconnectomics (cCCTomics) as a strategy for efficient and conditional targeting of chemical transmission
Source: eLife. 2024 Apr 30;12:RP91927. doi: 10.7554/eLife.91927 (PMC11060718; doi:10.7554/eLife.91927)
Supplement: Figure 3—source data 3. [file elife-91927-fig3-data3.zip › Figure 3 source data3 part 1.zip!/Figure 3-Source data 3-part1/1067-9-CRISPResso_Report_c33kf3u3.zip!/CRISPResso_on_1067_10/1a.Read_barplot.pdf]

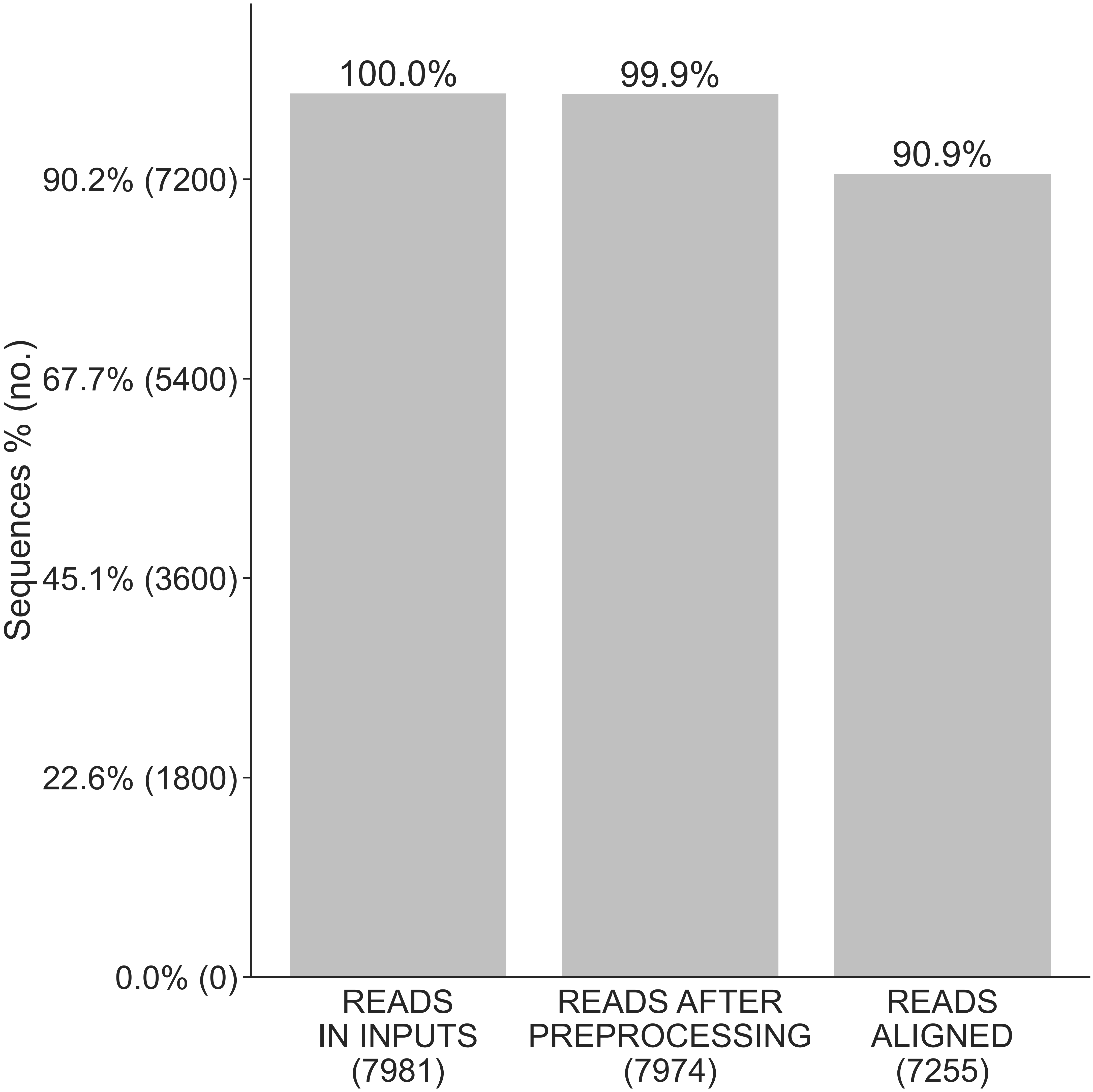

100.0%
99.9%
90.9%
90.2% (7200)
67.7% (5400)
45.1% (3600)
22.6% (1800)
0.0% (0)
Sequences % (no.)
READS
IN INPUTS
(7981)
READS AFTER
PREPROCESSING
(7974)
READS
ALIGNED
(7255)
